# Adopt or Adapt: Sanitation Technology Choices in Urbanizing Malawi

**DOI:** 10.1371/journal.pone.0161262

**Published:** 2016-08-17

**Authors:** Richard M. Chunga, Jeroen H. J. Ensink, Marion W. Jenkins, Joe Brown

**Affiliations:** 1 Department of Infectious and Tropical Diseases, London School of Hygiene and Tropical Medicine, London, United Kingdom; 2 Department of Civil and Environmental Engineering, University of California at Davis, Davis, California, United States of America; 3 School of Civil & Environmental Engineering, Georgia Institute of Technology, Atlanta, United States of America; Cardiff University, UNITED KINGDOM

## Abstract

This paper presents the results of a mixed-methods study examining adaptation strategies that property owners in low-income, rapidly urbanizing areas in Malawi adopt to address the limitations of pit latrines, the most common method of disposing human excreta. A particular challenge is lack of space for constructing new latrines as population density increases: traditional practice has been to cap full pits and simply move to a new site, but increasing demands on space require new approaches to extend the service life of latrines. In this context, we collected data on sanitation technology choices from January to September 2013 through 48 in-depth interviews and a stated preference survey targeting 1,300 property owners from 27 low-income urban areas. Results showed that property owners with concern about space for replacing pit latrines were 1.8 times more likely to select pit emptying service over the construction of new pit latrines with a slab floor (p = 0.02) but there was no significant association between concern about space for replacing pit latrines and intention to adopt locally promoted, novel sanitation technology known as ecological sanitation (ecosan). Property owners preferred to adapt existing, known technology by constructing replacement pit latrines on old pit latrine locations, reducing the frequency of replacing pit latrines, or via emptying pit latrines when full. This study highlights potential challenges to adoption of wholly new sanitation technologies, even when they present clear advantages to end users. To scale, alternative sanitation technologies for rapidly urbanising cities should offer clear advantages, be affordable, be easy to use when shared among multiple households, and their design should be informed by existing adaptation strategies and local knowledge.

## Background

Africa is predominantly rural but rapidly urbanising. In many cities, over 70% of the urban residents reside in low-income and highly dense informal or semi-formal communities [1]. These areas are characterised in part by lack of basic services, uncertain or legally unrecognised property rights, poverty, and unhealthy living conditions [2]. Pit latrines (a hole in the ground with a slab floor, a small squat hole and enclosed in a shelter) are generally the most common form of sanitation in these settings, rather than sewerage. Pit latrines eventually fill up and must be replaced or emptied, and where space is available the simplest solution is to cover the full pit, dig a new one, and move or construct a new superstructure. Urbanization and increasing population density make this practice unsustainable, creating the need for new technologies or adaptation of existing technologies (including better faecal sludge management (FSM), to extend the service life of latrines [3]. Pit emptying in these settings is challenging because latrines may not be designed to be emptied, satisfactory pit emptying equipment may not be available, and pit emptying vehicles may not be able to access sites where these services are needed [4,5]. Pit emptying, often via manual scooping with buckets or adding water to facilitate pumping, may lead to a variety of potential exposures among pit emptiers as well as those living where faecal sludges are disposed [5,6], often close to the pit since transport and disposal options for faecal sludges may be limited. To support property owners to maintain access to sanitation and reduce environmental contamination from untreated faecal sludge, non-governmental organisations have introduced ecological sanitation in urbanizing communities of Malawi [7,8].

Ecological sanitation (ecosan) is an alternative sanitation technology that offers a different approach to the operation and the maintenance of decentralised sanitation. A key feature of ecological sanitation facilities is that they are designed to be emptied and human excreta is regarded as a resource to be recycled and used as fertiliser for food crop production rather than waste to be disposed [9]. Its operation includes an explicit focus on reduction of disease risk through composting faecal sludges, a process which raises the temperature of waste over time to inactivate a wide range of enteric pathogens, if well managed. In addition to reducing risks, proponents of ecological sanitation argue that the technology is ideal where space for replacing pit latrines is limited or where the construction of pit latrines is challenging because of shallow bedrock or high groundwater table [8,10]. Although the potential advantages of ecosan in these settings would seem to suggest the technology may be widely adopted, widespread and persistent local promotion since 2000 has resulted in only very limited uptake. We undertook this study to understand (1) why ecosan uptake has been low in this context, and (2) how communities are meeting the challenge of increasing demands on space in sanitation technology choice. We collected data through mixed methods research, starting with a series of in-depth interviews followed by a stated preference survey. We hypothesized that increasing urbanization pressure and related constraints on space are driving specific sanitation choices in this context.

## Materials and Methods

### Study site and sanitation infrastructure

We conducted this study in Lilongwe and Blantyre City in Malawi. The National Statistics Office (NSO) reported that in 2008, Lilongwe City had a population of 669,021 people, an annual population growth rate of 4.3% and a population density of 1,479 persons per square kilometre while Blantyre City had a population of 661,444 people, an annual population growth rate of 2.8% and a population density of 3,006 persons per square kilometre [11]. In Lilongwe City, 22% of the population are classified as poor while in Blantyre City, 7.5% are classified as poor [12].

The majority of residents in low-income urban areas are tenants who depend on sanitary facilities provided by their landlords, generally property owners. Tenants have little say with respect to the type and quality of sanitation facilities on offer; further, they may not be empowered to demand sanitation improvements or be willing to contribute toward costs for sanitation improvements or maintenance as temporary residents lacking property rights [13]. In study sites at the time of this survey, about 90% of the residents used dry pit latrines while 10% used flush sanitation facilities connected to a septic tank or sewer system. Pit latrines here are usually over 2 meters deep and on average, they are used for a mean of 3.9 years before being replaced [14]. Based on Joint Monitoring Programme definitions [15], it is estimated that 50% of the residents in urban areas in Malawi use improved sanitation (facilities that ensure hygienic separation of human excreta from human contact e.g. pit latrines with a slab), 45% use shared sanitation (sanitation facilities shared by two or more households), 3% use unimproved sanitation (sanitation facilities that do not ensure hygienic separation of human excreta from human contact e.g. pit latrines lacking a cleanable slab), and 2% do not have access to sanitation [16].

Ecosan was introduced in urban areas in Malawi in 2000 by non-governmental organisations (NGOs). Two types of ecosan have been locally promoted: the urine diverting toilet (UDT) and the fossa alterna (FA). A UDT has two vaults constructed above ground, used alternately [8]. Urine and faeces are collected separately, using a urine-diverting squat plate. Users are instructed to add dry matter (ash, soil, and/or sawdust) into the vault after defecating to reduce odours and to enhance composting; urine is collected in a suitable plastic bucket or directed into a soak pit. When one vault is full, it is covered and left to mature and the second vault is used. When the second vault is full, the contents of the first vault–now posing low risks in handling and disposal–are emptied and users revert to the first vault. The removed pit contents may be applied as fertilizer. An FA toilet has two shallow pits (each 1.5 m deep) which are also used alternately [8]. In the FA, urine is not separated from faeces.

The adoption of ecosan in urban areas has been very slow. The social welfare monitoring survey conducted by the National Statistics Office in 2011 showed that only 0.2% of urban residents in Malawi were using ecosan [17]. At the time of data collection, Centre for Community Organisation and Development (non-governmental organisation at the forefront of ecosan promotion) estimated that there were about 4,000 ecological sanitation facilities in the two cities (verbal communication).

Other than introducing ecosan, NGOs have also introduced gulpers for pit emptying. A gulper is a manually operated pump for extracting faecal sludge from pit latrines [18]. At the time of data collection, there were two contractors emptying pit latrines with gulpers in Blantyre City and one individual in Lilongwe City. Faecal sludge was often emptied into 200 litre drums and transported to treatment stations on pick-up trucks; it was also common for contractors to dispose of faecal sludge adjacent to the emptied pit.

### Data collection

#### Qualitative study

We carried out a qualitative study in low income urban areas where WaterAid and Water for People (Non-governmental organisations) were implementing water supply, sanitation and hygiene interventions. Data collection started with a series of unstructured, in-depth interviews (IDIs). We selected respondents using purposive convenience sampling [19] to understand perceptions of ecosan, factors causing property owners to become concerned about space for replacing pit latrines, and local adaptation strategies to urbanization pressures. Respondents included property owners that were currently using ecosan (adopters), property owners that were not using ecosan (non-adopters), and tenants using ecosan. Among non-adopters, we included respondents who used both improved and unimproved sanitation facilities. Survey questions were open-ended and intended to collect detailed information on space and population density pressures and sanitation technology adoption. We continued to conduct interviews until additional interviews failed to add new information or themes [19]. We triangulated information collected from property owners and tenants with information collected from builders and hygiene promoters. The builders and hygiene promoters were invited to attend focus group discussions (FGDs). Hygiene promoters were volunteers supporting WaterAid and Water for People in sanitation and hygiene promotion.

#### Analysis of qualitative data

We digitally recorded and manually transcribed interviews and FGDs. Data analysis involved the following four steps: (a) listening to digital recordings and reading transcripts to list recurrent themes, (b) identifying key themes by which the data generated was examined, (c) developing a thematic framework and (d) rearranging the data according to the appropriate part of the thematic framework [20]. The second step produced a range of possible causes of concern about space for pit latrines, adaptation strategies, factors motivating property owners to adopt ecosan, and barriers preventing property owners from adopting ecosan. For each respondent, we recorded identified causes of concern about space for replacing pit latrines, adaptation strategies identified, positive attributes (motivating factors), and negative attributes of ecosan. Qualitative data collection served the primary purpose of informing the development of survey instruments for use in the larger stated preference surveys to follow.

### Stated preference survey

The qualitative study was followed by a stated preference survey. We sampled low-income, high-density urban areas from a list of such areas prepared by the Lilongwe and Blantyre City Councils under the Participatory Slum Upgrading Programme [21,22]. We calculated the sample size to include a representative sample of property owners concerned about space for replacing pit latrines, estimated at 26% of property owners based on a formative research and according to standard formulae [23], assuming sampling error of 5%, a clustering design effect (DE) of 2, and an increase of 10% to account for non-response. The resulting sample size was 1300 property owners located in 27 neighbourhoods across the two cities.

We used a two-stage sampling technique to select respondents. In the first stage, we selected low-income urban areas based on probability proportion to population. In the second stage, research assistants sampled property owners randomly by starting from a central point and selecting every 5^th^ house until they interviewed a pre-specified number of property owners [24]. We excluded property owners using septic tanks or sewerage since a primary focus of the survey was pit emptying.

We collected the following data using a semi structured questionnaire: type of pit latrine in use, number of houses at a property, access to a garden for food crop production, type of domestic water source, income status, gender of property owner, availability of a vacant space at a property and knowledge of alternative sanitation technology including ecosan. We also examined the level of concern about space for replacing pit latrines, high groundwater table, and shallow bedrock using a 4-point Likert scale.

To examine sanitation technology choices, we offered property owners a range of sanitation options: pit emptying service, pour flush toilets, ecosan (UDTs and FA), and pit latrines with and without improvements such as slabs or lined pits. Property owners were informed that they were free to select any technology of their choice; we used photographs to describe both technology choices and pit emptying options, explaining the advantages, disadvantages and estimated costs before asking respondents to select the technology of their choice (S1 File).

#### Data analysis

We used EpiData version 3.1 and Stata version 12 to process and analyse the data, beginning with descriptive statistics. We used binary logistic regression analysis to examine the characteristics of property owners that were concerned about space for replacing pit latrine, reporting odds ratios, and used multinomial logistic regression analysis to examine whether concern about space for replacing pit latrines was associated with preference for ecosan. The likelihood of selecting ecosan was indicated by the Relative Risk Ratio (RRR) adjusted for income status, knowledge of ecological sanitation, number of households at a property, access to a garden for food crop production, type of sanitation facility in use and source of domestic water. In the multinomial logistic regression model, pit latrines with a slab floor were used as the reference category as the most common technology choice in this setting. For both the binary logistic regression and multinomial logistic regression, we conducted a series of univariable analyses to select variables to include in the final models. We included variables that had a p-value of 0.20 or less as independent variables in the full models [25].

### Ethics Approval

We obtained ethics approval from The London School of Hygiene and Tropical Medicine and from the National Health Research Council in Malawi. Research participants provided written consent to participate in surveys and FGDs.

## Results

### Qualitative study

We carried out 48 IDIs and 3 FGDs. Respondents to the IDIs included 9 property owners that had adopted ecosan, 6 tenants that were using ecosan, 20 property owners that were using improved sanitation, and 13 property owners that were using unimproved sanitation. Of the 48 respondents, 20 were men and 28 were women. A total of 27 hygiene promoters (6 male and 21 female) and 12 builders (11 male and 1 female) participated in FGDs.

**Fig 1:**
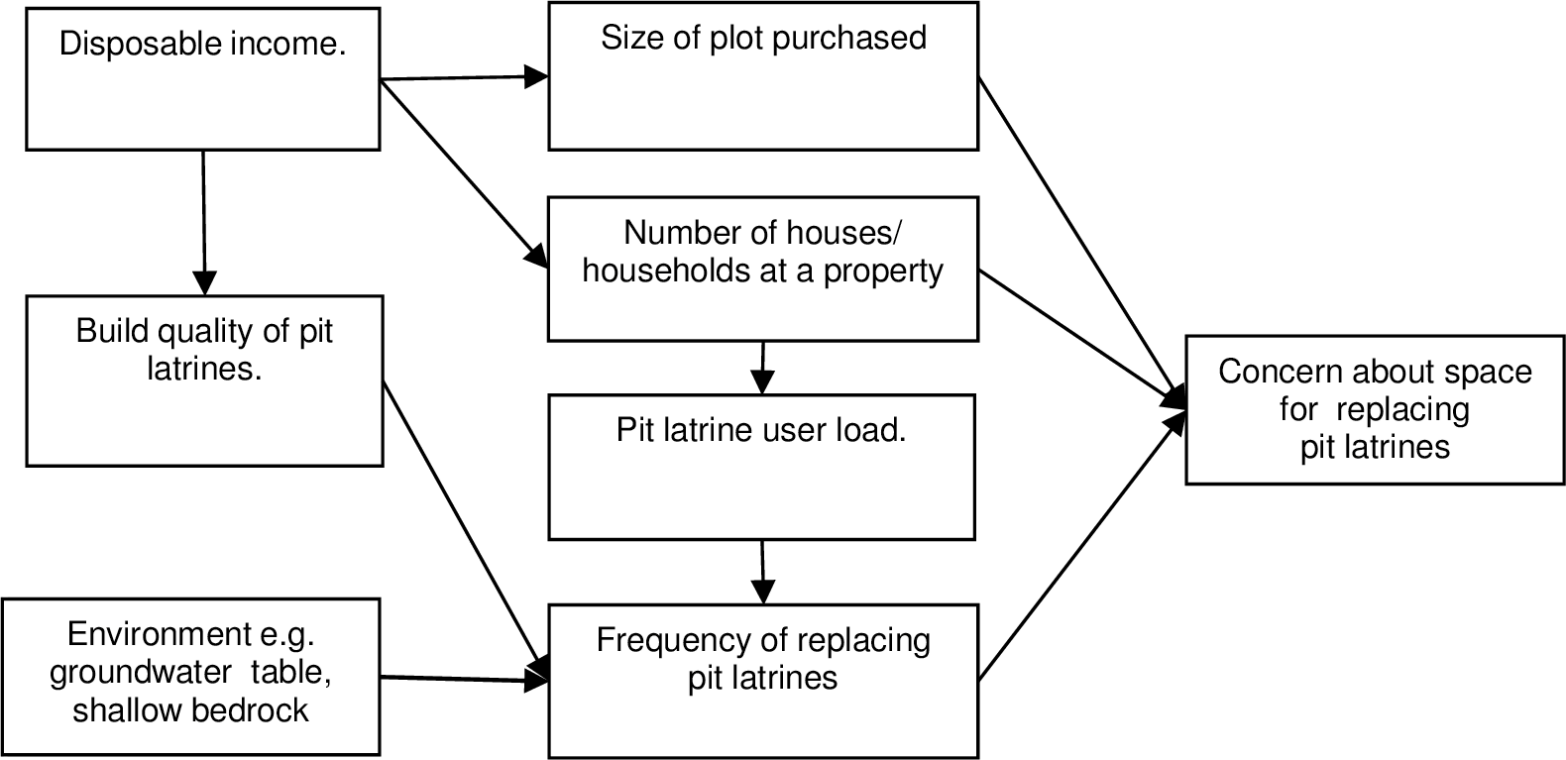
Key causes of concern about space for replacing pit latrines.

Respondents identified three key factors that drive concern about space for replacing pit latrines. These three factors include the size of plot/property purchased, the number of houses/households at a property and the frequency of replacing pit latrines. These factors are influenced by other factors as summarised in Fig 1.

Respondents explained that concern about space for replacing pit latrines arise when property owners replace pit latrines frequently (identified by 22 respondents). Pit latrines are replaced frequently because of their poor build quality (e.g. no roofing to protect the structure, floor constructed from timber and mud not cement), high pit latrine user load, and due to shallow bedrock or a high groundwater table, where it is more difficult to build pits and pits are more prone to collapse, respectively. Respondents further explained that concern about space for replacing pit latrines arises when property owners build multiple houses to increase rent revenue (identified by 17 respondents) and when they purchase relatively small plots due to low disposable incomes (identified by 16 respondents). Respondents also indicated that concern about space for replacing pit latrines arise when property owners build relatively big houses on small plots. Each of these factors is exacerbated by increasing demand for space and increasing population density, linked to rapid urbanization.

### Adaptation strategies

Through the IDIs, we identified four possible adaptation strategies that property owners pursue when there is concern about space for replacing pit latrines. Table 1 summarises the key adaptation strategies and possible actions under each adaptation strategy, as considered by respondents and indicated in FGDs.

**Table 1:** Adaptation strategies for decentralised sanitation in response to space constraints.

Adaptation strategy	Possible actions taken
1. Reducing the frequency of replacing pit latrines.	*(a) Improving the build quality of pit latrines* ■ Lining latrine pits with bricks and cement ■ Digging deep(er) pits ■ Roofing pit latrines ■ Adopting pit latrines with a slab floor
	*(b) Changing operational and maintenance practices perceived to prevent bulking or build-up of faecal sludges* ■ Adding water into pit latrines ■ Adding salt into pit latrines ■ Adding chemicals into pit latrines
2. Identifying an alternative space for pit latrine replacement	■ Constructing replacement pit latrines on an old pit latrine or bathroom location
3. Emptying pit latrines	■ Emptying pit latrines using local contractors
4. Alternative sanitation technologies.	■ Adopting ecosan ■ Adopting other alternative technologies

#### Adapting by reducing the frequency of replacing pit latrines

Where there is concern about space for replacing pit latrines, property owners adapt by reducing the frequency of replacing pit latrines. This is achieved by improving the build quality of pit latrines to prevent them from collapsing or filling up within a short time (identified by 19 respondents) or changing pit latrine operational and maintenance practices (identified by 11 respondents). Changing pit latrine operational and maintenance practices involves adding water (identified by 8 respondents), chemicals (identified by 2 respondents), or adding salt (identified by 1 respondent) into pit latrines. One respondent explained the strategy of adding water into pit latrines as follows: *“I will direct water from the bathroom to go into the pit latrine*. *The faeces dissolve and the toilet takes many years before filling up*. *Last time*, *it took 12 years*.*”*

#### Adapting by identifying alternative spaces for replacement pit latrines

Another adaptation strategy involves constructing replacement pit latrines on disused pit latrine spots or bathroom spots (identified by 13 respondents). Respondents explained that property owners usually avoid constructing replacement pit latrines on old pit latrine locations, since these sites are more likely to collapse as the soil is too loose to support the weight of the floor and superstructure. Where there is no other new space for constructing a replacement pit latrine, property owners may be forced to build replacement pit latrines on old pit latrine locations, despite this disadvantage. One respondent explained this strategy as follows: *“After some years*, *if there is no any other new space for a replacement pit latrine*, *we go back to old pit latrine locations*. *There is no problem because the faeces on old pit latrine locations become like compost*.*”* Alternatively, property owners build replacement pit latrines on a bathing room/shower room spot (identified by 3 respondents), which are generally separate from latrine sites. In low-income urban areas, bathing/shower rooms are usually built on plot but outside the house. When there is concern about space for replacing pit latrines, property owners build replacement pit latrines on bathroom spots as was explained by one respondent as follows: *“people pull down bathrooms and build replacement pit latrines on the bathroom spot and then convert the full toilet into a bathroom*.*”*

#### Adapting by emptying pit latrines

Respondents explained that when there is concern about space for replacing pit latrines, property owners adapt by emptying pit latrines when they fill up (identified by 10 respondents). Property owners usually empty pit latrines manually e.g. by scooping faecal sludge with buckets, digging a pit next to the full pit latrine and connecting the empty pit to the full pit through a hole so that some of the faecal sludge should flow into the empty pit, often assisted by the addition of water.

#### Adapting by adopting alternative sanitation technologies

Respondents explained that property owners also adapt by adopting new technologies, specifically ecosan. (identified by 3 respondents). Respondents were attracted to the concept of ecosan due to three main factors. Respondents perceived ecosan facilities to be permanent (identified by 29 respondents) and easier to empty (identified by 25 respondents). One respondent explained the advantage of adopting ecosan as follows: “*The advantage of ecological sanitation is that it is for life*, *there will be no need to construct another sanitation facility*.*”* Another respondent explained this positive attribute as follows: *“it is forever—even your granddaughters and grandsons will use the same latrine*.*”* Respondents were also attracted to the concept of ecosan because it offers users access to fertiliser for food crop production (identified by 22 respondents).

Through the IDIs, we identified three key negative attributes or barriers that prevent or would prevent property owners from adopting ecosan. These barriers included operation and maintenance challenges (identified by 31 respondents), incompatibility with multiple households sharing facilities (identified by 18 respondents), affordability or cost constraints (identified by 13 respondents) and disgust with handling human excreta from ecological sanitation facilities (identified by 5 respondents). Commenting on the challenges of operating and maintaining ecosan, one property owner explained the disadvantage of the technology as follows: *“Tenants say that it is too involving to be adding ash and soil*. *I will construct another pit latrine simply because my tenants say that they cannot manage to operate and maintain ecological sanitation*. *However*, *I don’t know how we are going to construct another pit latrine as there is a problem of space for pit latrines here*.*”* A tenant who had experience in using ecosan explained the difficulties of operating and maintaining ecosan: *“I think ecological sanitation is not ideal where there are multiple households because people do not take care of the facility*. *If the landlord would allocate ecological sanitation to one household*, *I would accept to use it but not that all of us at this property should be using one ecological sanitation facility*.*”* One property owner explained his dissatisfaction with ecosan as follows: “*I am saying that one needs a pit latrine because ecological sanitation facilities fill up within six months so if you have tenants*, *the ecological sanitation facility will be filing up very quickly so it is better to build a pit latrine for tenants*.*”* Commenting on the installation cost of ecological sanitation, one property owner explained that: “*These toilets are very helpful but we cannot afford them because we also need to buy food*.*”*

### Stated Preference Survey

We interviewed 1300 property owners. Descriptive statistics from the sample are presented in Table 2. Ecosan use was rare as only 2% were using UDTs and only four property owners were using FA at the time of sampling. When asked about concern about space for replacing pit latrines, 16% indicated that they were very concerned about space for replacing pit latrines, 6% were somewhat concerned and 3% were little bit concerned. Thus there was concern about space at 25% of the properties sampled.

**Table 2:** Descriptive statistics (n = 1300).

Variable	*N*	*%*	*Mean*	*Max*	*Std*
*Type of sanitation facility in use*					
Pit latrine with a slab/cement floor	658	51			
Pit latrines with mud floor	363	28			
Lined pit latrine	227	17			
Urine diverting toilet *(ecological sanitation)*	28	2			
Fossa alterna toilet *(ecological sanitation)*	4	0			
Pour flush toilet	1	0			
No sanitation facility	19	1			
*Shared sanitation facility*					
Yes	819	63			
No	481	37			
*Availability of vacant space at the property*					
Yes	958	74			
No	342	26			
*Concern about space for replacing pit latrines*					
Not concerned at all	971	75			
Little bit concerned	41	3			
Somewhat concerned	80	6			
Very concerned	208	16			
*Education of property owner*					
No education	61	5			
Primary school	543	42			
Secondary school	588	45			
College	108	8			
*Gender of property owner*					
Male	971	75			
Female	329	25			
*Access to a garden for food crop production*					
Yes	447	34			
No	853	66			
*Prior Knowledge about ecological sanitation*					
Yes	891	69			
No	409	31			
Number of households at a property			3	15	2
Number of people at a property			11	56	7

### Characteristics of property owners concerned about space

Table 3 identifies four conditions that were associated with concern about space for replacing pit latrines: (1) a unit increase in the number of houses at a property increased the odds of a property owner indicating that he/she was concerned about space for replacing pit latrines (OR = 1.1, p = 0.01); (2) property owners who did not have vacant spaces compared to those who had vacant spaces within their property were 3.6 times more likely to indicate that they were concerned about space, (3) property owners that were using pit latrines with a slab floor compared to property owners that were using lined pit latrines were 2.4 times more likely to be concerned about space for replacing pit latrines and (4) property owners that were concerned about high groundwater table compared to those that were not concerned were 1.7 times more likely to be concerned about space for replacing pit latrines.

**Table 3:** Conditions associated with concern about space for pit latrines (n = 1198).

Variable	*N*	OR	p-value	95% Conf. int
Number of houses at a property		1.1	0.01	1.0–1.2
*Vacant space available*				
Yes (ref)	267			
No	931	3.6	0.00	2.7–4.5
*Type of current facility*	* *			
Lined pit latrine (ref)	220			
Pit latrine, slab floor	633	2.4	0.0	1.6–3.6
Pit latrine, mud floor	345	1.8	0.0	1.2–2.8
*Concern about high groundwater table*	* *			
No	924			
Yes	274	1.7	0.00	1.2–2.3
*Concern about shallow bedrock*	* *			
No (ref)	692			
Yes	506	1.1	0.35	0.9–1.5
*Income of property owner*	* *			
<K20,000 (ref)	391			
MK20,000–30,000	415	0.9	0.56	0.7–1.3
>MK40,000	392	0.9	0.48	0.6–1.2
model constant		0.2	0.00	0.1–0.4

Notes: Unadjusted results from binary logistic regression. Data exclude property owners with pour flush (1), ecological sanitation (32) and no sanitation facilities (19).

Sanitation technology choices where there is concern about space for replacing pit latrines

**Fig 2:**
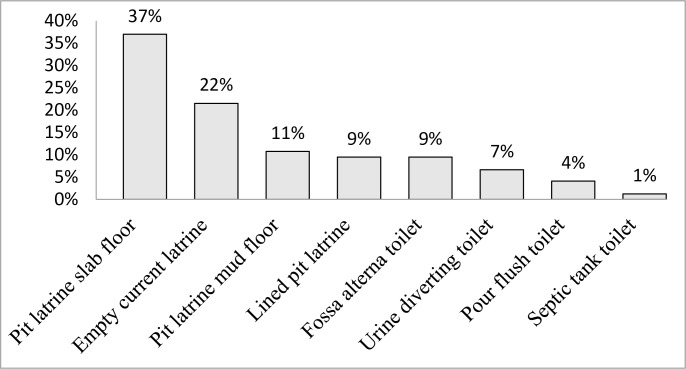
Technology choices of property owners concerned about space (n = 316).

Fig 2 shows the options that property owners that were concerned about space for replacing pit latrines preferred. Nine percent (30) of the property owners that were concerned about space for replacing pit latrines preferred FA toilets and 7% (21) preferred UDTs. Thus 16% (51) of the property owners that were concerned about space for replacing pit latrines preferred ecosan. Fewer property owners (4%) preferred pour flush toilets and only 1% preferred septic tank toilets while the remaining 79% preferred to empty their current pit latrine or install new pit latrines.

Table 4 examines the likelihood of selecting ecosan in this sample. Property owners stating concern about space for replacing pit latrines were 1.8 times more likely to select pit emptying service over the construction of new pit latrines with a slab floor (p = 0.02) but there was no significant association between concern about space for replacing pit latrines and intention to adopt ecosan as a potentially more permanent solution. The table further shows that income status and type of sanitation facility in use played a key role in the choices that property owners made. With regard to the income status of property owners, the results show that property owners in the first income category (poorer) compared to those in the third category (wealthier) were less likely to select ecosan over pit latrines with a slab floor (RRR = 0.6, p = 0.02) and poorer property owners compared to wealthier property owners were less likely to select water-based technologies (pour-flush) over pit latrines with a slab floor (RRR = 0.2, p < 0.001).

**Table 4:** Likelihood of selecting ecological sanitation over pit latrines when there is concern about space for pit latrines (n = 1198).

Variable	*n*	Septic tank /pour flush (Water based)		Ecological sanitation		Lined pit latrine		Pit emptying service		Pit latrine without slab floor(mud floor)	
		RRR	95% Conf.int	RRR	95% Conf.int	RRR	95% Conf.int	RRR	95% Conf.int	RRR	95% Conf.int
*Concern about space*											
No (ref)	891										
Yes	307	1.1	0.6–2.0	1.5	1.0–2.2	0.6*	0.4–0.9	1.8*	1.1–2.8	0.7	0.4–1.2
*Monthly income*											
>MK40,000 (ref)	392										
<MK20,000	391	0.2***	0.1–0.4	0.6*	0.3–0.9	0.6*	0.4–1.0	0.3***	0.2–0.5	3.8***	1.8–8.1
MK20,00–40,000	415	0.2***	0.1–0.4	0.6*	0.4–0.9	0.8	0.5–1.3	0.6*	0.4–1.0	1.7	0.8–3.7
*Type of sanitation in use*	* *										
Lined pit latrine (ref)	220										
Pit latrine, slab floor	633	0.03***	0.0–0.1	0.03***	0.0–0.2	0.01***	0.0–0.0	0.002***	0.0–0.0	0.03*	0.0–0.3
Pit latrine, mud floor	345	0.02***	0.0–0.1	0.06***	0.0–0.3	0.03***	0.0–0.2	0.0	0.0–0.0	1.5	0.2–11.3
Number of houses at a property		0.9	0.8–1.1	1.0	0.9–1.1	1.1	1.0–1.2	0.9	0.8–1.0	1.0	0.9–1.1
*Access to a garden*											
No (ref)	792										
Yes	406	0.7	0.4–1.4	1.4	1.0–2.1	1.1	0.7–1.7	0.8	0.5–1.2	1.2	0.8–1.9
*Source of water*											
Public water point	776										
Standpipe on the yard	422	2.1*	1.1–3.9	0.8	0.5–1.2	1.7*	1.1–2.5	0.8	0.5–1.2	0.6	0.3–1.1
*Knowledge of ecological sanitation*											
No (ref)	392										
Yes	806	0.6	0.3–1.1	1.2	0.8–1.5	1.0	0.7–1.5	1.0	0.6–1.6	0.8	0.5–1.3
Model constant		14.0*	2.5–77.8	9.3*	1.9–45.0	16.6***	3.6–76.6	167.8***	36.6–769.9	0.6	0.1–5.4

*Notes*: Results from multinomial logistic regression using pit latrines with a slab as the reference category. The sample (n = 1198) excluded respondents with ecological sanitation (32), no sanitation facility (19) and pour flush toilet (1). Data from 51 respondents were excluded because of inconsistency.

***p<0.001

*p<0.05

## Discussion

All pit latrines eventually fill up and must be replaced or emptied. Where there is no space for replacing pit latrines, property owners in this setting often empty pit latrines using unhygienic methods and dispose faecal sludge untreated into the open environment [3,13]. Untreated faecal sludge pollutes the environment and exposes urban residents to infection and disease [26]. Ecosan has been introduced in several settlements as an alternative sanitation technology that can reduce environmental pollution from faecal sludge and as a more sustainable solution as space becomes more constrained for replacing pits [8,26]. We examined motivations and stated preferences for sanitation technology decision-making in the context of urbanization and increasing population density. The results showed that when there is concern about space for replacing pit latrines, property owners preferentially adapt, mainly through non-technological strategies, rather than investing in wholly new technology. The adaptation strategies that property owners adopt have important implications on the design and promotion of alternative sanitation technologies in low-income and high population density urban areas, where many new technologies, products, and services are being developed to meet the sanitation and FSM needs of urban and urbanizing communities.

### Factors causing concern about space for replacing pit latrines

Previous work has suggested that space for replacing pit latrines may not be available when property owners build multiple houses at the expense of space for replacing pit latrines and when they do not use lined pit latrines [13,27]. The advantage of lined pit latrines is that they reduce the frequency of replacing pit latrines as they are less likely to collapse and can safely be emptied [3]. Our results confirm that respondents in our sample perceived these benefits. A unit increase in the number of houses/households at a property increased the odds that a property owner would indicate that he/she was concerned about space for replacing pit latrines and property owners that were using pit latrines with a slab floor and pit latrines with the floor made from mud or soil were more likely to be concerned about space for replacing pit latrines than property owners that were using lined pit latrines (Table 4). The results further suggest that property owners indicating concern about a high water table compared to those that were not concerned and property owners that did not have vacant spaces compared to those that had vacant spaces were more likely to be concerned about space for replacing pit latrines.

Results suggest that environmental challenges (high groundwater table, shallow bedrock) and the choices that property owners make (using unlined pit latrines, building multiple houses at the expense of space for sanitation) have significant implications on the availability of space for replacing pit latrines. To reduce environmental pollution from untreated faecal sludge as cities rapidly urbanise, city authorities and change agents should consider improving pit emptying services and influencing the way property owners build houses and sanitation facilities. Realistic policy options may include regulating the number of houses per plot/property and further promotion of latrine designs that are easier and safer to empty.

### Adaptation

The concept of adaptation originates from population biology and ecological sciences; applied to human systems, it may refer to a deliberate change in anticipation of or in reaction to external stress [28]. It has been observed that when there is a threat to individual wellbeing, individuals are likely to see the need for change and can and do adapt to changing environmental circumstances based on their experience, knowledge and available resources [29,30]. Research shows that a great deal of useful information is contained in indigenous knowledge systems and that when this information is dismissed, change agents introduce ideas that are unlikely to be successful [31].

### Adaptation of existing pit latrines

Non-technological adaptation strategies that property owners adopt are consistent with the idea that individuals adapt to their changing environmental conditions based on their knowledge, experience, and available resources [32]. Key adaptation strategies include: improving the build quality of pit latrines or changing pit latrine operational and maintenance practices to reduce the frequency of replacing pit latrines, emptying pit latrines when they fill up, and constructing replacement pit latrines on old pit latrine spots or bathroom spots.

The construction of replacement pit latrines on old pit latrine locations was the most frequently identified non-technological adaptation strategy. By constructing new pit latrines on old pit latrine locations, many property owners are already emptying their pit latrines without requiring vacuum tankers, manually operated faecal sludge emptying equipment or alternative sanitation technologies that need frequent emptying—as is the case with ecological sanitation. The average lifespan of pit latrines in Malawi is 3.9 years [14]. Therefore, the strategy of constructing new pit latrines on old pit latrine spots means that many property owners wait for several years before emptying and handling human excreta. In contrast, ecosan facilities in the targeted areas are emptied within 6 to 12 months depending on the number of users. Thus ecosan facilities are perceived as requiring more time and effort to manage.

While it is important to collect, treat and safely dispose or reuse human excreta in agriculture, the reality is that many cities in Sub-Saharan Africa do not currently have the financial resources and the infrastructure to undertake safe FSM [33]. Furthermore, vacuum tankers or manually-operated faecal sludge emptying equipment may not be effective in emptying pit latrines several years after they fill up as the sludge may become too thick [18]. City authorities and sanitation engineers should therefore explore how the practice of constructing new pit latrines on old pit latrine locations or on bathroom locations can be improved to be safer and more sustainable. The promotion of removable latrine superstructures (shelters) and slabs could make the process of constructing new pit latrines on old pit latrine locations easier. The promotion of removable bathrooms should also be considered. These options are worth exploring because they would allow property owners to store human excreta onsite until it may be safer to handle the excreta.

### New technologies

The promotion of ecosan in low-income and high population density urban areas offers property owners an opportunity to address the limitations of pit latrines through the adoption of this technology (technological adaptation) [10]. Besides access to cheap fertiliser for food crop production, property owners were attracted to the concept of ecosan because they perceived the technology to be permanent and easier to empty. However, few property owners had intention to adopt ecological sanitation as an adaptation strategy and, for this context, ecosan represents a promising but ultimately failed intervention strategy. Our results confirm that individuals usually respond to their changing environmental circumstances mainly through incremental behavioural adjustment rather than through transformational change (e.g. adoption of new technologies) because of the effort and financial resources required to implement transformational actions [34]. Among property owners that were concerned about space for replacing pit latrines, few property owners (16%) preferred ecosan and fewer (5%) preferred pour flush or septic tank toilets while the majority (79%) were intending to empty pit latrines or construct new pit latrines. The results suggest that the current design and promotion of ecological sanitation in this setting is not meeting the needs of most property owners that are concerned about space for pit latrines.

### Barriers to technological adaptation

#### Affordability

Sanitation research has shown that people adopt alternative sanitation technologies when there are no barriers preventing them from adopting the alternative technologies [35]. Several researchers have explained that the adoption of ecosan is slow because it is too expensive for the poor [10,36]. Our results support this observation. Poorer property owners compared to wealthier property owners were less likely to adopt ecosan. At the time of the study, a property owner needed MK70, 000 to 90, 000 (USD 155–200) to purchase a UDT and MK30, 000 to 50, 000 (USD 67–110) to adopt an FA toilet. Considering that a slab for a pit latrine was only about MK5, 000 (USD 11) and pit latrine emptying was MK20, 000 (USD 44), it was cheaper to adapt by emptying pit latrines or improving the build quality of replacement pit latrines to extend their lifespan (e.g. adopting pit latrines with a slab) than adopting ecosan in this context.

#### Complexity of ecological sanitation and its compatibility to the needs of property owners

Users of ecosan are expected to add dry matter (ash and soil or saw dust) into the chambers that collect faecal matter after defecating and they are expected to empty the facilities more frequently than pit latrines (6). Research about acceptance and diffusion of innovations has shown that technologies that are perceived to be complex to use and not compatible with the needs of users are less likely to be adopted [31,37]. The task of adding dry matter and emptying ecosan is perceived to be too involving particularly when the technology is shared among multiple households [38]. Other researchers have observed low levels of satisfaction with the technology and low use at plots/properties where users have also access to pit latrines [39]. Our results are consistent with these observations.

Although ecosan offers property owners concerned about space for replacing pit latrines important advantages (easier to empty, no need to replace the facility), the technology is less likely to be adopted because it is not affordable and it is too involving to use. This study underscores a central emerging theme in sanitation innovation research: proposed technologies must meet the needs of target population and be easy to operate and maintain, especially when shared among multiple households. This is consistent with the theory of diffusion of innovation [31,40]. The use of shared sanitation is widespread, with an estimated 784 million users of “shared public” and “shared private” latrines globally [16]. In the sampled areas, 63% of the sampled properties had shared sanitation. The design and promotion of alternative sanitation technologies must therefore be informed by this reality.

### Addressing root causes of lack of space for replacing pit latrines

As urban populations increase, property owners are likely to continue building additional houses within their properties to generate money through rent [13]. Ecosan offers property owners with limited space for replacing pit latrines a potential solution. However, the promotion of ecosan does not address the root cause of the problem of space for replacing pit latrines. To be sustainable, solutions to environmental challenges must also aim at addressing the underlying causes of undesired change [41,42]. One of the key causes of the problem of lack of space for replacing pit latrines is the tendency of property owners to build multiple houses at the expense of space for replacing pit latrines. The results showed that 74% of the sampled property owners had vacant spaces and could build an additional house if they wanted to. Considering that local governments in Malawi, like those in many other countries in Sub-Saharan Africa, do not currently have the capacity to safely collect, treat and dispose faecal sludge from pit latrines, it is important that local governments consider regulating the number of houses property owners build to ensure that those with vacant spaces reserve adequate space for replacing pit latrines.

### Limitations

This study and our conclusions from it should be qualified with the known limitations of the data collection and analysis. First, our work on ecosan and demand for ecosan may be specific to this intervention, the local programming that has supported its dissemination, and the local context, and therefore our conclusions about the advantages of adaptation over adoption of new technology may not be generalizable to other technologies in other settings. Second, several questions about pit emptying services were based on the gulper, a locally promoted and manually operated pit emptying tool, which does not empty entire pits. Focusing pit emptying questions on the gulper may have affected the choices of property owners who had prior knowledge of the gulper and pre-existing opinions about its usefulness. Lastly, this study was based on stated preferences from interviews and not revealed demand or preferences through market data. Stated preferences may differ significantly from actual preferences, demand, and behaviours. Other stated preference surveys give respondents time (a couple of days) to think about their options, which can have advantages. Due to limited time and resources of this large sample, survey respondents were not given time to think about their options, though interviewers did take time to explain the options and answer questions from respondents.

## Conclusion

Urbanization and associated increasing population density exert pressures on sanitation infrastructure, creating incentives for property owners to adapt existing technology or adopt new technology. After years of ecosan promotion, low adoption and low stated preference for adoption in urbanizing areas suggest its advantages as a more permanent solution do not outweigh its perceived disadvantages in this context. Instead, property owners prefer to adapt pit latrines to respond to increasing demands on space, as modifications are cheaper and perceived to be more compatible with the way property owners and their tenants have traditionally been operating and maintaining sanitation facilities. Alternative sanitation technologies designed and promoted to address the limitations of pit latrines in low-income and high population density urban areas should offer clear advantages and be affordable and easy to use, particularly when shared among multiple households as is common in like settings. In addition, their design should be informed by existing adaptation strategies as innovative technologies will be evaluated against the existing strategies.

## Supporting Information

S1 File Product information provided to survey respondents. (DOCX)

S2 File Survey data. (XLSX)
