# Supplementary material for: Adopt or Adapt: Sanitation Technology Choices in Urbanizing Malawi
Source: PLoS One. 2016 Aug 17;11(8):e0161262. doi: 10.1371/journal.pone.0161262 (PMC4988694; doi:10.1371/journal.pone.0161262)
Supplement: S1 File — (DOCX) [file pone.0161262.s001.docx]

## Product information provided to survey respondents

| Technology | Advantages presented to survey respondents | Disadvantages presented to survey respondents | Estimated cost offered to survey respondents  in Malawi Kwacha (MK) |
| --- | --- | --- | --- |
| Urine diverting toilet (UDT) | - Access to compost - Permanent facility (build once never build again) - Will not collapse. - Safer for children. - Does not smell. - Make you look modern. - Cheaper to empty. | - Shallow depth. - Empty regularly. - Collecting ash and soil - Adding ash and soil after defecating. - Handling compost from the toilet. | 70,000 to 90,000 MK  (155 – 200 USD) |
| Fossa alterna | - Access compost - Permanent facility. - Will not collapse. - Safer for children. - Does not smell. - Make you look modern. - Cheaper to empty. | - Shallow depth. - Empty regularly. - Collecting ash and soil - Adding ash and soil after defecating. - Handling compost from the toilet. | 30,000 to 50,000 MK  (67 – 111 USD) |
| Pour flush toilet | - Permanent facility. - Less likely to collapse. - Does not smell. - Make you look modern. | - Need money to empty it when it fills up. - Must have access to water for flushing. | 90,000 to 110,000 MK  (200- 244 USD) |
| ***Lined pit latrine***. Research assistants introduced a lined pit using a photograph. The advantages and disadvantages of lined pit latrines were not discussed as was the case with the alternative sanitation technologies. It was assumed that survey respondents were familiar with the concept of lining pits.  these are familiar | | | 70,000 to 90,000 MK  (155 – 200 USD) |
| ***A pit latrine slab***. The advantages and disadvantages of a slab were not discussed as was the case with alternative sanitation technologies. It was assumed that survey respondents were familiar with the concept of a pit latrine with a slab considering that pit latrines with a slab floor were common in the study area. | | | 5,000 MK  (11 USD) |
| Emptying pit service using a gulper. Respondents were shown pictures of men emptying a pit latrine using a gulper. Respondents were informed that people emptying with a gulper are able to reach houses even where there are no roads for vacuum tankers. Survey respondents were not informed about how deep their facilities would be emptied. The service was based on the assumption that the entire pit would be emptied and that the pit emptiers would empty the faecal sludge at a treatment plant. | | | 20,000 MK (44 USD) |
| In addition to these options, survey respondents were informed that they could install any other sanitation technology of their choice including pit latrines without slab floor (unimproved sanitation) and septic tanks. It was difficult to estimate the installation cost of pit latrines without slab floor as property owners use a range of materials and sometimes construct pit latrines on their own. The installation cost of a septic tank (flush toilets) was also not offered as it was assumed that property owners were knowledgeable about septic tanks. | | |  |

*1 United States Dollar (USD) = 450 Malawi Kwacha (MK) at the time of the survey*
